# Molecular Mechanisms, Biomarkers and Emerging Therapies for Chemotherapy Resistant TNBC

**DOI:** 10.3390/ijms23031665

**Published:** 2022-01-31

**Authors:** Paola Ferrari, Cristian Scatena, Matteo Ghilli, Irene Bargagna, Giulia Lorenzini, Andrea Nicolini

**Affiliations:** 1 Unit of Oncology 1, Department of Medical and Oncological Area, Pisa University Hospital, 56126 Pisa, Italy; 2 Division of Pathology, Department of Translational Research and New Technologies in Medicine and Surgery, University of Pisa, 56126 Pisa, Italy; cristian.scatena@unipi.it; 3 Unit of Breast Surgery, Breast Cancer Center, University Hospital of Pisa, 56126 Pisa, Italy; m.ghilli@ao-pisa.toscana.it; 4 Unit of Medical Oncology, Department of Translational Research and New Technologies in Medicine, University of Pisa, 56126 Pisa, Italy; irene.bargagna@hotmail.it (I.B.); lorenzinigiuli@gmail.com (G.L.); andrea.nicolini@med.unipi.it (A.N.)

**Keywords:** breast cancer, triple-negative, chemoresistance, biomarkers, emerging therapies

## Abstract

Triple-negative breast cancer (TNBC) is associated with high recurrence rates, high incidence of distant metastases, and poor overall survival (OS). Taxane and anthracycline-containing chemotherapy (CT) is currently the main systemic treatment option for TNBC, while platinum-based chemotherapy showed promising results in the neoadjuvant and metastatic settings. An early arising of intrinsic or acquired CT resistance is common and represents the main hurdle for successful TNBC treatment. Numerous mechanisms were uncovered that can lead to the development of chemoresistance. These include cancer stem cells (CSCs) induction after neoadjuvant chemotherapy (NACT), ATP-binding cassette (ABC) transporters, hypoxia and avoidance of apoptosis, single factors such as tyrosine kinase receptors (EGFR, IGFR1), a disintegrin and metalloproteinase 10 (ADAM10), and a few pathological molecular pathways. Some biomarkers capable of predicting resistance to specific chemotherapeutic agents were identified and are expected to be validated in future studies for a more accurate selection of drugs to be employed and for a more tailored approach, both in neoadjuvant and advanced settings. Recently, based on specific biomarkers, some therapies were tailored to TNBC subsets and became available in clinical practice: olaparib and talazoparib for *BRCA1/2* germline mutation carriers larotrectinib and entrectinib for neurotrophic tropomyosin receptor kinase (*NTRK*) gene fusion carriers, and anti-trophoblast cell surface antigen 2 (Trop2) antibody drug conjugate therapy for heavily pretreated metastatic TNBC (mTNBC). Further therapies targeting some pathologic molecular pathways, apoptosis, miRNAS, epidermal growth factor receptor (EGFR), insulin growth factor 1 receptor (IGF-1R), and androgen receptor (AR) are under investigation. Among them, phosphatidylinositol 3 kinase (PI3K)/protein kinase B (Akt)/mammalian target of rapamycin (mTOR) and EGFR inhibitors as well as antiandrogens showed promising results and are under evaluation in Phase II/III clinical trials. Emerging therapies allow to select specific antiblastics that alone or by integrating the conventional therapeutic approach may overcome/hinder chemoresistance.

## 1. Introduction

Triple-negative breast cancer (TNBC) is defined as a tumor lacking estrogen (ER) and progesterone (PR) receptor expression and human epidermal growth factor receptor 2 (HER-2) overexpression/amplification. TNBC represents 10–20% of breast cancers and is more frequent in young women [1]. As compared to that of the other breast cancer (BC) subtypes, TNBC is associated with higher incidence of recurrence and distant metastases, and shorter overall survival (OS) [2]. Despite better pathological complete response (pCR) rates after neoadjuvant chemotherapy, prognosis of TNBC patients is worse as compared to non-TNBC tumors; this phenomenon is known as “triple negative paradox” [3]. In TNBC patients, disease progression and recurrence typically occur within the first 3–5 years after diagnosis; brain and lung metastases are more common [2,4]. This behavior is attributed to higher biological aggressiveness, including the emergence of resistance to chemotherapy (CT), which is the mainstay treatment in TNBC. In fact, although chemoresistance is shared with most other malignancies, an intrinsic origin or an earlier occurrence is much more common in this molecular subtype. TNBC is usually diagnosed by immune-histochemistry (IHC).

Basing upon gene expression patterns, five molecular subtypes of breast cancer with distinctive clinical behavior were identified, i.e., Luminal A, Luminal B, Her-2 enriched, Normal-like, and Basal-like [5,6]. Among them, basal-like breast cancers are most commonly triple-negative. However, these two terms are not synonymous, as 70–80% of TNBCs are basal-like and about 70% of basal-like cancers are triple-negative [6]. More recently, a TNBC subgroup termed claudin-low molecular subtype was identified. This subtype lacks basal markers and is enriched in stem cell and epithelial–mesenchymal transition (EMT) markers [7]. Overall, these findings underline the heterogeneous nature of TNBC. 

In early-stage TNBC, various rates of pCR after neoadjuvant chemotherapy (NACT) as well as different response to treatment and different survival in the metastatic setting were found [8]. Tumor heterogeneity and multiple mechanisms of chemoresistance may be largely responsible for this phenomenon [9,10]. The molecular heterogeneity of TNBC was better clarified by genomic sequencing studies. In particular, basal-like 1 (BL1), basal-like 2 (BL2), immunomodulatory (IM), mesenchymal (M), mesenchymal stem-like (MSL), and luminal androgen receptor (LAR) molecular subtypes were identified [9]. A further classification into four subtypes was made by Burstein et al.: androgen receptor (AR) positive, mesenchymal, basal-like immune sup-pressed, and basal-like immune activated [10]. These subtypes might predict response to targeted therapy; however, they are not used in clinical practice, and cytotoxic chemotherapy remains the mainstay in TNBC treatment. This review, after having briefly examined the chemotherapeutic regimens recommended by current guidelines in the different settings, focuses on the main mechanisms reported to be responsible of chemoresistance in TNBC. Successively, biomarkers helpful in predicting resistance to chemotherapy and drugs either currently recommended or potentially useful in chemoresistant TNBC are considered.

## 2. Chemotherapy

CT is currently the principal therapeutic option in TNBC. Preferred regimens include taxanes and anthracyclines; platinum-based regimens are also used in neo-adjuvant and metastatic settings [11].

### 2.1. Neo-Adjuvant Setting

Current guidelines recommend as preferred regimens: doxorubicin (or epirubicin)-cyclophosphamide combination (dose dense or every 3 weeks) followed by paclitaxel with or without carboplatin; docetaxel-cyclophosphamide combination; olaparib if germline *BRCA1/2* mutation; addition of pembrolizumab in high-risk patients [12,13].

Despite the aggressive nature of TNBC, 20% of patients achieve a pCR after NACT [14]. However, TNBC patients who did not achieve pCR are more likely to suffer an early recurrence and die from metastatic disease. The differences in clinical outcomes following neo-adjuvant treatment imply that a subset of TNBCs is sensitive to CT, while the majority become resistant during treatment or are intrinsically less susceptible. Both mechanisms are likely present in the tumors. However, in early, operable TNBC, the administration of CT before (neoadjuvant) rather than soon after surgery (adjuvant) was considered an optional strategy. Therefore, the role of pCR as a treatment endpoint and a guide for further treatment decisions is considered crucial, and it becomes not an option but rather the preferred treatment strategy for TNBC breast cancer patients [15].

### 2.2. Adjuvant Setting

Current guidelines recommend as preferred regimens: doxorubicin (or epirubicin)-cyclophosphamide combination (dose dense or every three weeks) followed or not by paclitaxel; docetaxel-cyclophosphamide combination; and cyclophosphamide-methotrexate-5-fluorouracil (CMF) combination. Recently, the CREATE X clinical trial showed that, in TNBC breast cancer patients, postsurgical capecitabine can improve prognosis when the disease persists after NACT [12,13].

### 2.3. Metastatic Setting

The main drugs used in this setting include anthracyclines, platinum compounds, taxanes, capecitabine, gemcitabine, vinorelbine, eribulin; sacituzumab govitecan; bevacizumab; olaparib and talazoparib for *BRCA1/2* germline mutations carriers; addition of atezolizumab or pembrolizumab to chemotherapy for pogrammed death ligand 1 (PD-L1) overexpressing cancer patients; larotrectinib and entrectinib for neurotrophic tropomyosin receptor kinase (NTRK) gene fusion carriers [12,13].

## 3. Main Mechanisms Responsible for Chemoresistance

CT resistance represents a main obstacle for successful cancer treatment, especially in the metastatic setting where it accounts for 90% of therapy failure [16]. In the last decade, numerous mechanisms were uncovered that can lead to the development of chemoresistance. These include cancer stem cells (CSCs) induction after NACT, ATP-binding cassette (ABC) transporters, hypoxia and avoidance of apoptosis, tyrosine kinase receptors (EGFR, IGFR1), a disintegrin and metalloproteinase 10 (ADAM10), noncoding RNAs, DNA methylation, and phosphoproteome, including phosphorylation of kinases and a few pathological molecular pathways.

### 3.1. CSCs Induction after NACT 

In solid tumors, CSCs represent a subpopulation with self-renewal properties, that can re-establish a tumor following treatment. In breast cancer, an increase in CSCs was found in residual tumor specimens following CT [17,18]. This finding suggests that breast CSCs are resistant to treatment; moreover, their selective survival may lead to a residual tumor enriched in tumor-initiating cells. Accordingly, a high percentage of CSCs in primary breast tumors following NACT was found [19]. TNBC seems to be enriched in CSCs as compared with that of other subtypes [20,21]. Some data seem to support the importance of CSCs in TNBC behavior and prognosis. For example, a positive correlation between the expression of stem cell markers (CD44, ALDH1) and poor survival was found [22,23]. Chemoresistant CSCs may be important in TNBC relapse. In TNBC biopsies carried out following CT, upregulation of RNA transcripts of CSCs associated genes was found [24]. Treatment of TNBC cells with gemcitabine or paclitaxel stimulated the expression of hypoxia-inducible factors (HIFs), as well as the increase in CSCs population and ABCB1 expression [25]. Mechanisms responsible for CSC chemoresistance are still unclear. CSCs are relatively quiescent as compared to that of other cancer cells, and this behavior could be a defense against cytotoxic agents that are generally most effective against proliferating cells [17]. Moreover, CSCs have high ABC transporters expression, mostly ABCG2, which confers resistance to many cytotoxic agents [26,27]. 

### 3.2. ABC Transporters

Transporter-mediated drug efflux is one of the most studied mechanisms of chemoresistance. ABC transporters are integral membrane proteins responsible for the ATP-powered translocation of various compounds across cellular membranes, including many anticancer drugs [28]. Multidrug-resistant protein-1 (ABCC1/MRP1), breast cancer resistance protein (ABCG2/BCRP) and multidrug-resistant protein-8 (ABCC11/MRP8) are significantly more expressed in TNBC as compared to that of other subtypes [29]. It was observed that NACT increased ABCC1 protein expression in TNBC [30]. In addition, TNBC cell activation of the hedgehog pathway increased drug resistance through upregulation of ABC transporters [31]. ABCG2 is involved in TNBC CSCs, and its downregulation increased chemosensitivity in TNBC cells [26,32]. ABCC1 confers resistance mainly to anthracyclines, taxanes, mitoxantrone, and methotrexate; ABCG2 transports 5-Fluorouracil, methotrexate, doxorubicin, irinotecan, mitoxantrone, and other drugs [28]. ABCC11 confers resistance to 5-Fluorouracil and methotrexate [33]. In conclusion, ABCC1, ABCG2, and ABCC11 have a wide and overlapping substrate specificity and may confer resistance to the principal antiblastics used in the current TNBC treatment.

### 3.3. Hypoxia

As the tumor expands, blood vessels randomly grow and are often cut-off or destroyed. Low oxygen levels lead to HIFs stabilization; HIFs regulate transcription of genes involved in cell survival in hypoxic conditions. Hypoxia is an important feature of the tumor microenvironment (TME) and is associated with aggressiveness, invasiveness, and resistance to therapy [34]. Hypoxia contributes to chemoresistance in multiple different ways: firstly, insufficient vasculature hinders drug penetration [34]; in addition, the acidic TME due to hypoxia reduces the uptake of anticancer drugs [35]; cytotoxic effects of drugs are often oxygen dependent [36]; hypoxia induces the CSC phenotype [37]; hypoxia activates immunosuppressive pathways and acts as a barrier to immune effector cells, thereby modulating antitumor immunity [38]; hypoxia leads to cellular adaptations that hinder a successful treatment, for example, increased expression in ABC transporters, and decreased proliferation; modulation of apoptosis, induction of autophagy, genetic instability, and subsequent selection of aggressive phenotypes, upregulation of proangiogenic factors, and repression of E-cadherin [36,39,40,41]. Morphological features typical of hypoxia, for example, fibrotic and necrotic areas, are frequently present in TNBC specimens [42]. Moreover, a study showed that the expression of carbonic anhydrase IX (*CAIX*), a key HIF-1 regulated gene, was associated with TNBC subtype and shorter survival [43].

### 3.4. Avoidance of Apoptosis

Apoptosis is a key biological process largely dysregulated in cancer disease and evasion of apoptosis takes part of the main cancer hallmarks. Evasion of apoptosis also was reported to be responsible of resistance to different cytotoxic agents, such as paclitaxel, doxorubicin, and cyclophosphamide [44]. The role of apoptotic dysfunction in TNBC prognosis is well documented. For example, expression of pro-survival factors, such as Bcl-2 and Mcl-1, was found to be related to unfavorable outcome [45,46]. In addition, *MCL-1* gene is frequently altered in residual TNBCs after CT [47]. A link between Mcl-1 expression and chemoresistance was reported [48]. So, Mcl-1 expression likely contributes to TNBC chemoresistance [45].

### 3.5. Receptor Tyrosine Kinases

Multiple biological processes are due to different growth factors using PI3K-AKT-mTOR (PAM) and janus kinase (JAK)/signal transducer and activator of transcription (STAT) signaling pathways. EGFR and IGF-1R, which are part of the receptor tyrosine kinase family, are the upstream regulators of these pathways implicated in TNBC chemoresistance.

#### EGFR, IGF-1R 

**Figure 1:**
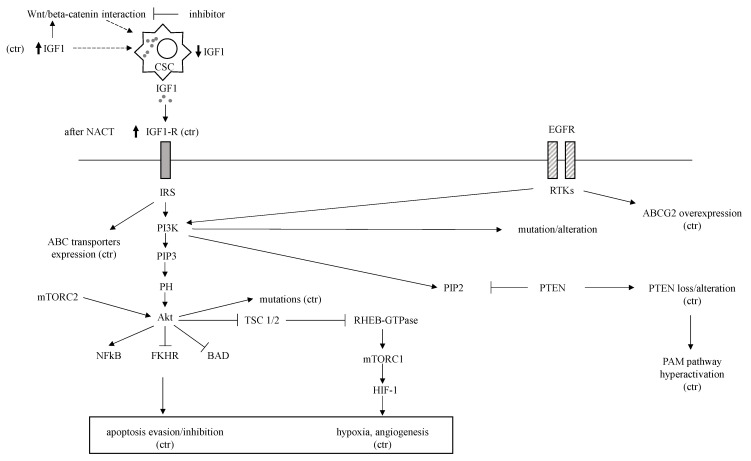
EGFR, IGF1-R, and PI3K-Akt-mTOR (PAM) pathway. Potential mechanisms of chemotherapy resistance (ctr) in TNBC. PI3K activation produces PIP3 from PIP2 substrate; Akt activation inhibits TSC, which acts as a GTPase activating protein for RHEB; mTORC1 induces hypoxia and angiogenesis via modulation of HIF-1; mTORC2 promotes apoptosis evasion/inhibition through NFkB, FKHR, and BAD; PTEN and TSC are significant tumor suppressors. IGF1: insulin growth factor 1; IGF1-R: insulin growth factor 1 receptor; CSC: cancer stem cell; NACT: neoadjuvant chemotherapy; EGFR: epidermal growth factor receptor; RTKs: receptors with protein tyrosine kinase activity; PIP2: phosphatidylinositol-4,5 biphosphate; PIP3: phosphatidylinositol-3,4,5 triphosphate; PH: protein with pleckstrin homology; Akt: protein kinase B; GTPase: guanosine triphosphatase; HIF-1: hypoxia inducible factor 1; IRS: insulin receptor substrate; mTORC1/2: mammalian target of rapamycin complex 1/2; PI3K: phosphatidylinositol 3 kinase; PTEN: phosphatase and tensin homolog deleted on chromosome 10; TSC: tuberous sclerosis; RHEB: RAS homolog enriched in brain (GTP-binding protein); FKHR: forkhead family transcriptor factors; ABC: ATP binding cassette; ABCG2: ATP binding cassette superfamily G member 2; NFkB: nuclear factor kappa-high chain enhancer of activated B cells; BAD: Bcl-2 associated death promoter. ↑ increase; ↓ decrease. Also see text.

EGFR overexpression in TNBC ranges from 13–76% and is higher than in other breast cancer subtypes [6]. *EGFR* gene amplification correlates with protein expression and seems to be the most crucial mechanism behind increased EGFR expression in TNBC [49]. The EGFR pathway is involved in the regulation of ABCG2 expression and function [50]; in fact, EGFR inhibition led to the reversal of ABCG2-mediated chemoresistance in in vitro and tumor models [51]. EGFR pathway is also involved in angiogenesis, cell proliferation, metastatic spread, and inhibition of apoptosis [51]. Insulin-like growth factors (IGFs) binding to their receptor results in proliferation, expression of ABC transporters, angiogenesis stimulation and inhibition of apoptosis [52]. Expression of IGF-1R in TNBCs was found in up to 46% of cases and was associated with poor survival [53]. It was observed that IGF-1 interacts with the wingless and Int1 (Wnt)/beta-catenin pathway and is overexpressed CSCs in TNBC [54]. Beta-catenin inhibition reduced IGF-1 levels, and this led to decreased CSCs proliferation [54]. IGF-1R overexpression is linked to chemoresistance in various types of cancer [52]. Finally, it was found that IGF-1R was overexpressed in breast tumors following NACT and was associated with shorter OS [55]. These findings are summarized in Figure 1.

### 3.6. A Disintegrin and Metalloproteinase 10 (ADAM10)

One study investigated the potential roles of ADAM10 on TNBC cells and the effects of combining ADAM10 expression and NACT to improve the OS in breast cancer patients. Knockdown of ADAM10 in MDA-MB-231 cells led to a significant decrease in cell proliferation, migration, invasion, and the IC50 value of paclitaxel and adriamycin, while also inducing cell cycle arrest and apoptosis. These changes were correlated with downregulation of Notch signaling, CD44, and cellular prion protein (PrPc). Immune-histochemical staining for ADAM10 in breast cancer tissues of 94 patients receiving NACT also was performed. A high ADAM10 expression in pre-NACT samples was strongly associated with poorer response to NACT and shorter OS. These data suggest that ADAM10 plays an important role in contributing to the progression and chemoresistance of TNBC [56].

### 3.7. Noncoding RNAs, DNA Methylation, and Phosphoproteome including Phosphorylation of Kinases

Epigenetic remodeling through noncoding RNAs of gene expression profiles and alterations of DNA methylation play a central role in the pathogenesis, maintenance, and therapeutic resistance of TNBC. NcRNAs constitute about 98% of the genome, and others include microRNAs (miRNAs), long noncoding RNAs (lncRNAs), and circular RNAs (circ RNAs) [57]. In recent years, despite these RNAs not coding proteins, a lot of data supported the central role of miRNAs and lncRNAs in chemoresistance by governing different genes and molecular pathways. When gene is the target, they function as oncogenes or tumor suppressor genes to lastly induce or inhibit chemoresistance. Aberrant expression of miRNAs and lncRNAs and subsequent promotion of apoptotic resistance, EMT, TME disorder, and ABC transporters upregulation are some modalities by which both these ncRNAs favor the occurrence of chemoresistance. Additionally, aberrant expression of miRNAs can promote cell cycle arrest, CSCs, DNA repair, autophagy inhibition, and thereafter, chemoresistance. CircRNAs interact with miRNAs, thus providing a novel mechanism to promote chemoresistance. Namely, it was shown that circCDR1as induces chemoresistance to 5-FU in TNBC by inhibiting miRNA-7 and successively governing cyclin E1 (CCNE1), while circKDM4C, which is downregulated in DOX resistant TNBC cells, favors DOX resistance by inhibiting cell cycle and apoptosis; this inhibition promotes the oncogenic action of miRNA548p, and subsequently, PBLD (phenazine biosynthesis-like domain containing protein) degradation [58]. 

DNA methylation is governed by the balance of DNA methylases (DNMTs) and DNA demethylases (TETs), and TNBCs are characterized by strong hypomethylation and lower gains of methylations compared to that of all other subtypes. In a study [59], whole-genome DNA methylation profiling on diagnostic TNBC biopsy samples from the Sequential Evaluation of Tumors Undergoing Preoperative (SETUP) NACT study was carried out. At the time of diagnosis, nine significantly differentially methylated regions (DMRs) were associated with response to NACT, and four of them also were associated with TNBC OS (*p* < 0.05). In another investigation [60], the percent DNA methylation ratio (PMR) of paired-like homeodomain transcription factor 2 (PITX2) was assessed by a validated methylation-specific real-time PCR test. Tissue samples and clinical data from 66 untreated and 78 TNBC patients treated with anthracycline-based CT were analyzed. PITX2 DNA methylation with a PMR cutoff of two did not show significance for poor vs. good outcomes (OS) in the untreated patient cohort (HR = 1.55; *p* = 0.259). By contrast, the PITX2 PMR cutoff of two identified patients with poor (PMR > 2) vs. good (PMR ≤ 2) outcomes (OS) with statistical significance in the anthracycline-treated cohort (HR = 3.96; *p* = 0.011). In a further recent study [61] conducted on TNBC, five differentially methylated sites (DMSs) signatures with good performance for the prediction of DFS and OS were constructed. 

Activation of protein kinases and phosphatases through phosphorylation is involved in signal transduction, and the basal level of the phosphoproteins is a main feature of the cell. In a study [62], high-throughput technology was used to evaluate changes in phosphorylated proteins to identify relevant pathways involved in TNBC chemoresistance. Four resistant and two sensitive to docetaxel, doxorubicin, gemcitabine, and cisplatin cell lines were selected among 12 TNBC cell lines. A total of 1340 phosphoprotein groups, 2760 unique phosphopeptides, and 4549 unique phosphosites were defined. It was found that differentially phosphorylated cyclin-dependent kinase (Cdk) 5, promyelocytic leukemia protein (PML), protein 1(AP-1) transcription factor, and heat shock factor 1 (HSF-1) might collaborate to promote EMT in the drug-resistant cells, with EGFR and hormone growth factor (HGF) likely involved in this process. In the human genome there are about 538 kinase genes [63] and aberrant expression or activation of protein kinases also may be implicated in drug resistance. In a study, MDA-MB-231 cells were used to monitor cell viability and were screened with a kinome siRNA library to identify potential drug resistance kinases in TNBC cells treated with CT. Genetic or pharmacological ablation of the Src kinase decreased the phosphorylation of AKT and STAT3 and increased the sensitivity of TNBC cells to chemotherapeutic drugs. Concomitant overexpression of Src and STAT3 was associated with poor prognosis in TNBC patients. Authors concluded that Src inhibitor combined with chemotherapeutic drugs might be beneficial in Src-expressing TNBC patients [64,65].

### 3.8. Pathological Molecular Pathways

#### 3.8.1. TGF-Beta Pathway

Tumor growth factor (TGF)-beta takes part of a large cytokine superfamily that encompass over 30 related growth factors, including three TGF-beta isoforms (TGFbeta-1-3) [66]. EMT, proliferation, angiogenesis, metastatic spread, CT resistance, and immuno-modulation are described effects of TGF-beta signaling [67]. Besides, the TGF-beta pathway plays a crucial role for the regulation of breast CSCs [68]. In TNBC, it was reported that CT enhanced TGF-beta signaling [24]. Furthermore, a TGF-beta-R inhibitor in TNBC xenografts impeded the re-establishment of tumors following CT [24]. Both TGF-beta overexpression and elevated breast CSC markers were found in epirubicin-resistant TNBC cell lines [69]. 

#### 3.8.2. Notch Pathway

The Notch signaling pathway comprehends four cell surface receptors (NOTCH 1-4) and five trans-membrane ligands (Delta-like 1,3,4 and JAGGED-1,2). Notch 1-4 signaling plays a key role for the maintenance of breast CSCs and significantly correlates with resistance to CT [70]. In breast cancer cell lines, Notch-1 signaling induced by doxorubicin promoted ABCC1 overexpression. Conversely, gamma-secretase inhibitor (GSI) reverted the Notch-1 induced ABCC1 overexpression, and thus the cells became more susceptible to doxorubicin [71]. This effect also occurred in TNBC cells, where GSI increased the efficacy of doxorubicin [72]. Consistent with these findings, Notch-1 inhibitors synergized with docetaxel in TNBC and had robust antitumor action in breast CSCs and patient-derived xenograft models [73].

#### 3.8.3. Wnt/Beta-Catenin Pathway

**Figure 2:**
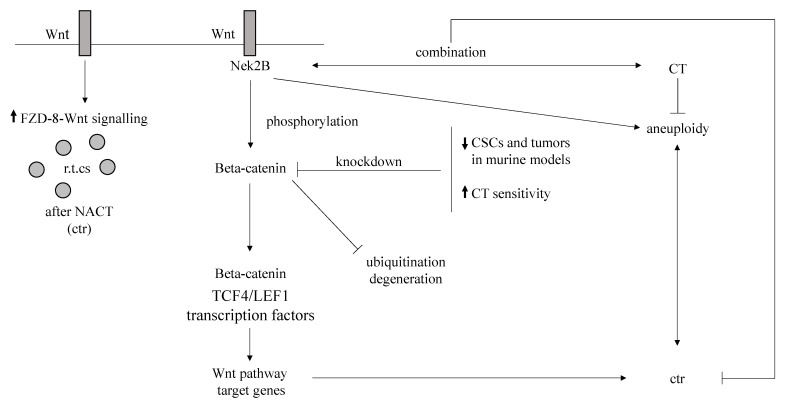
Wnt-beta-catenin pathway. Potential mechanisms of chemotherapy resistance (ctr) in TNBC. FZD-8: Frizzled-8; NEK2B: NIMA related kinase 2; CSCs: cancer stem cells; CT: chemotherapy; TCF: T cell factor; LEF: lymphocyte enhancer factor; r.t.cs: residual tumor cells; NACT: neoadjuvant chemotherapy. ↑ increase; ↓ decrease. Also see text.

Wnt signaling likely promotes tumor initiation, stemness, and metastatic spread [74,75]. In the lack of Wnt, beta-catenin quickly deteriorates following the action of the multi-protein destruction complex. Binding of Wnt to its receptors and co-receptors (Frizzled and low-density lipoprotein receptor-related proteins (LRP5/6), respectively) dissolves the destruction complex stabilizing beta-catenin. Accordingly, TNBC cells with knocked-down beta-catenin are highly susceptible to CT and form significantly smaller tumors in murine models. Wnt/beta-catenin signaling knock-down decreased the TNBC stem cell population [76]. Beta-catenin synergized with NIMA related kinase 2 (Nek2B) regarding CT resistance in TNBC [77]. An upregulation of other components of the Wnt/beta-catenin pathway was also found in TNBC, while FZD8-mediated Wnt signaling that was significantly enhanced in residual cells after NACT had a major role in TNBC chemoresistance [78]. The potential mechanisms of CT resistance are schematically shown in Figure 2.

#### 3.8.4. Hedgehog (Hh) Pathway

The Hh signaling pathway is a crucial network for embryogenesis and tissue regeneration. The Hh pathway includes three secreted ligands, of which the Sonic Hedgehog (SHH) is the most widely expressed, followed by trans-membrane receptor/co-receptors Patched (PTCH) and Smoothened (SMO). Activated SMO originates the full-length activator form of GLI transcription factors-GLIA. Three glioma-associated oncogene transcription factors (GLI1–3) are the principal effectors, and GLIAs, after they have moved to the nucleus, govern the expression of many target genes, such as *ABCG2* and *VEGF* (vascular endothelial growth factor) [79]. GLI1/2 are associated with cell survival, proliferation, invasion, EMT, angiogenesis, and chemoresistance in different human tumors [80]. Growing evidence suggests relationship of Hh signaling with more clinical aggressiveness of TNBC and Hh signaling is also strongly linked with CSC in TNBC. While both GLI1 and GLI2 are overexpressed in breast CSCs, cell differentiation significantly decreased their expression [81]. Hh signaling association with larger tumor size, high grade, high stage, and with poor prognosis in TNBC was reported [82]. In breast cancer cell lines, docetaxel treatment activated Hh signaling that increased survival and expanded breast CSC [81]. After exposure of malignant cells to cytotoxic drugs, GLI1 was overactivated via noncanonical pathway and, successively, promoted the ABC transporters upregulation [31].

#### 3.8.5. NF-kB Pathway

**Figure 3:**
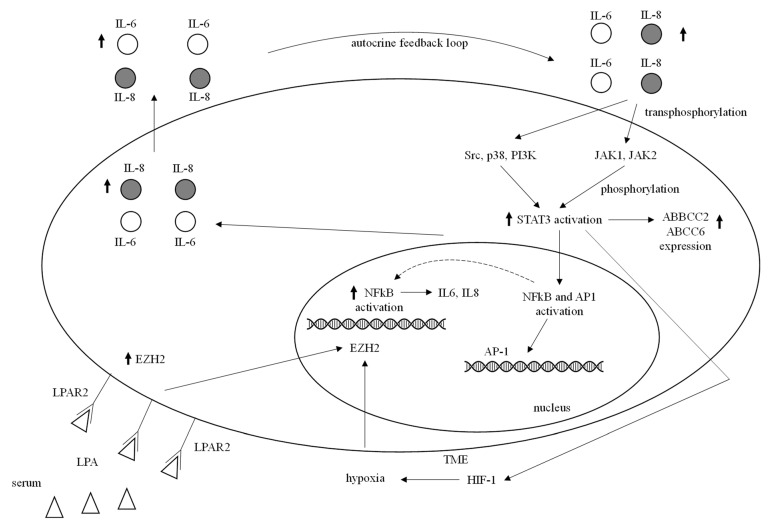
NFkB and JAK/STAT pathways. Potential mechanisms of chemotherapy resistance (ctr) in TNBC. NFkB is upregulated by hypoxia and activated through LPA-LPAR-EZH2-NFkB signaling cascade which results in autocrine production of IL-6 and IL-8. Extracellular IL-6, IL-8 ligand permits transphosphorylation of JAKs that successively phosphorylate STAT monomers. Activated STAT3 enters nucleus, where it governs transcription of many target genes; activated STAT3 also induces upregulation of ABCC2 and ABCC6, and therefore IL-6 and IL-8 by multiple pathways induce tumor growth, resistance to apoptosis, and chemotherapy resistance. LPA: lysophosphatidic acid; LPAR: LPA receptor; EZH2: enhancer of zeste homolog 2 (a gene component of polycomb repressive complex (PRC2) and epigenetic regulator); TME: tumor microenvironment; IL: interleukin; NFkB: nuclear factor kappa-high chain enhancer of activated B cells; STAT: signal transducer and activator of transcription; Src: Src family kinase; p38: p38 mytogen-activated protein kinase; PI3K: phosphatidylinositol 3 kinase. ↑ increase. Also see text.

NF-kB (nuclear factor kappa-light-chain-enhancer of activated B cells) family comprehends five members that can give origin to hetero- and homodimers [83]. NF-kB is strongly overexpressed in TNBC compared to that of normal breast tissue [84], and NF-kB activation promotes chemoresistance in breast and other types of cancer [83]. NF-kB signaling is also upregulated by hypoxia, which has a clear connection with chemoresistance [85]. In a recent report, it was found that apatinib sensitizes doxorubicin-resistant breast cancer cells to doxorubicin, which is accompanied by significantly increased apoptosis. The increased induction of apoptosis was associated with reactive oxygen species (ROS) accumulation, likely through the inhibition of NF-κB signaling pathways, which were showed to increase ROS production and reverse doxorubicin-resistance. Moreover, the combination of doxorubicin and apatinib resulted in increased antitumor effects on TNBC cell xenograft models [86]. Figure 3 shows the role of NFkB pathway in CT resistance in TNBC.

#### 3.8.6. PTEN and PI3K-AKT-mTOR Pathway

PAM pathway is frequently hyperactivated in TNBC, mainly due to the negative regulator tumor suppressor phosphatase and tensin homolog (*PTEN*) loss, and is associated with adverse clinical course, aggressive tumors, and poor outcome [87,88,89]. In TNBC, approximately 10% of patients have an activating mutation in *PIK3CA* and 30–50% with *PTEN* alterations [87]. Different subtypes of TNBC have specific phosphoinositide 3-kinase (PI3K) pathway mutations/alterations; for example, *PIK3CA* and *AKT1* mutations are more likely to be found in AR-positive TNBC [87]. *PTEN* loss also contributes to chemoresistance of breast cancer [90]. In addition, AKT induces HIF-1, which is a notable factor in chemoresistance [88] (Figure 1).

#### 3.8.7. JAK/STAT Pathway 

The JAK/STAT pathway encompasses four cytoplasmic proteins with Janus kinase domain (JAK1–3, TYK2) and seven proteins that comprise the signal transducer and activator of transcription protein family (STAT1–4, STAT5A, STAT5B, and STAT6). Extracellular interleukin (IL) 6, IL8 ligand allows the trans-phosphorylation of JAKs which then phosphorylate STAT monomers. Activated STATs enter the nucleus and subsequently govern the transcription of several target genes [91]. In TNBC, genetic profiling uncovered a pro-inflammatory gene signature including *IL6* and *IL8* genes [92]. Combined inhibition of IL6 and IL8 significantly induced apoptosis, and increased TNBC sensitivity to paclitaxel [92]. STAT3, a member of the JAK/STAT signaling pathway downstream from IL6/8, is overexpressed in TNBC and linked with tumor initiation, clinical aggressiveness, unfavorable outcome, and resistance to CT [93,94]. STAT3-NF-kB interaction and collaboration account for chemoresistance in TNBC [95]. Furthermore, *STAT3* was upregulated in TNBC stem cells resistant to doxorubicin [96]. *STAT3* was also involved in hypoxia-mediated chemoresistance in TNBC through *HIF1* upregulation. Interestingly, STAT3 further contributed to hypoxia-induced chemoresistance by upregulation of ABC transporters expression [97] (Figure 3). 

The principal reasons likely responsible for chemoresistance in triple-negative breast cancer are summarized in Table 1.

## 4. Prediction of Resistance to Chemotherapy in TNBC

CT is the mainstay of TNBC treatment and chemoresistance is a hurdle in neoadjuvant as well as in the metastatic settings. Histology and tumor-infiltrating lymphocytes (TILs) provide useful information in predicting resistance to CT. 

### 4.1. Histology and Molecular Subtype

Some studies reported a worse prognosis for metaplastic carcinoma, a rare, aggressive subtype of breast cancer associated with poorer OS than that of other TNBCs [98]. Particularly, the squamous subtype had the worst survival [99]. Metaplastic breast cancer, which was also reported as poorly responsive to neoadjuvant treatment [100], frequently expresses immune checkpoint markers forkhead box P3 (FOXP3) and PD-L1 and may benefit from immune-based therapies [101]. The principal TNBC subtypes [10,102,103] display varying levels of chemoresistance, which is reflected in their pCR rates after NACT [104]. The luminal androgen receptor (LAR) subtype is the most resistant subtype based on the information received from several clinical trials and preclinical studies [104,105]. It was observed that LAR tumors are relatively quiescent, which could partially explain their chemoresistance [106]. After LAR, the lowest pCR rates were observed in mesenchymal (MES) tumors [104]. TNBC cell cultures with mesenchymal properties, such as MDA-MB-231 and hs578t, display high levels of chemoresistance. The MES subtype of TNBC is enriched in gene expression signatures linked with EMT and stemness. The basal-like (BL1 and 2) group demonstrates high pCR rates; it is characterized by robust proliferation and is enriched in genes involved in cell cycle and DNA damage response [102]. *BRCA1/2* is frequently inactivated in BL1 subtype due to mutations or hyper-methylation. This leads to deficiencies in DNA damage repair, thus making these tumors more susceptible to DNA damaging agents [102]. 

### 4.2. Tumor Infiltrating Lymphocytes (TILs) and Neoadjuvant Response

TILs predict the NACT efficacy pre-, post-, or during treatment in the different molecular subtypes, mostly in TNBC. In the neoadjuvant setting, studies underlined the relevance of TILs evaluation for predicting pCR and TILS rate significantly correlated with a better TNBC and HER2-positive breast cancer prognosis [107]. In a study carried out in 1058 patients, in those with TIL infiltration more than 10% following neoadjuvant anthracycline/taxane-based CT, pCR rate was 40–42% compared to only 3–7% in patients with tumors with low TIL infiltration. Elevated TIL infiltration in TNBC correlated directly with pCR after neoadjuvant anthracycline CT alone, taxane-based regimens alone, and anthracycline and taxane sequentially or concurrently administered [108]. Similarly, an association between high TILs and pCR in a group of TNBC patients receiving paclitaxel followed by a combination of fluorouracil, epirubicin, and cyclophosphamide (FEC) occurred [109]. Recently, the international TILs Working Group, renamed the “International Immuno-Oncology Biomarker Working Group on Breast Cancer” elaborated an integrated survival prediction model for patients with early-stage TNBC. The model involved TILs, PD-L1 and Cluster of Differentiation 73 (CD73) expression in a tissue immune profile (TIP) [110]. A TIP positive (TIP+) tumor was any tumor with contemporaneous presence of TILS ≥ 50%, PD-L1 ≥ 1%, and CD73 ≤ 40%. Sixty biopsies from patients with TNBC who received standard NACT were retrospectively examined. pCR was achieved in 23 patients (38.0%), 12 (20.0%) of whom were TIP+. The pCR rate was significantly higher in TIP+ (91.7%) than in TIP− (25.0%) (*p* < 0.0001), and using a multivariate analysis, TIP was confirmed to be an independent predictive factor of pCR (OR 49.7 (6.30–392.4), *p* < 0.0001). The combined TIP was more accurate than single biomarkers in predicting pCR [111].

#### TIL Subsets

The infiltration of CD3+ T cells was reported to predict the response to NACT in breast cancer [112]. In TNBC patients receiving neoadjuvant anthracycline/anthracycline + taxane-based therapy, increased CD4+, CD8+, and FOXP3+ TIL infiltration correlated with pCR [113]. Similarly elevated pCR rates occurred in patients with high infiltration in pretreated biopsies of both FOXP3+ and CD8+ TILs who received neoadjuvant paclitaxel followed by FEC [109]. An increased CD8/FOXP3+ TIL ratio in pretreatment biopsies significantly correlated with pCR in TNBC and HER2-positive breast cancer, following FEC100 and paclitaxel + trastuzumab respectively. CD20+ TIL (B cells) significantly correlated with pCR and CD20 overexpression joined with a 5.5 times likelihood of a pCR to a neoadjuvant anthracycline + taxane combination [105]. A significant decrease in cytotoxicity of circulating natural killer (NK) cells was found in tumors poor responsive to NACT [114]. Conversely, a significant increase in NK cells in the peritumoral environment but not in intratumoral NK cells was associated with tumors having a good pathological response. Increased activity of NK cells in the peripheral blood after NACT joined with the disappearance of lymph node metastasis in breast cancer patients [115,116,117,118]. In patients with locally advanced breast cancer, elevated pre-NACT circulating neutrophils and their significant decrease concomitant with pCR in axillary lymph nodes with metastatic involvement after eight cycles of capecitabine, docetaxel, adriamycin, and cyclophosphamide neoadjuvant regimens were reported [119]. A significant increase in circulating dendritic cells was found in breast cancer patients whose tumors had a good pathological response after neoadjuvant regimens with adriamycin and cyclophosphamide followed by capecitabine and docetaxel. However, a significant decrease in the intratumoral CD1a + tumor-infiltrating DCs was shown, without any significant association with response to therapy, in both primary breast tumors and metastatic axillary lymph nodes [119].

### 4.3. Biomarkers Helpful in Predicting Chemoresistance 

In TNBC management, molecular testing can identify many different biomarkers capable to predict chemoresistance in general or chemoresistance to specific commonly used chemotherapeutic agents. They include BRCAness and deoxyribonucleic acid (DNA) homologous recombination deficiency (HRD), lnc RNAs, micro RNAs, circular RNAs, C-X-C motif chemokine ligand 8 (CXCL8)- C-X-C motif chemokine receptor (CXCR) 1/2 axis, different molecules (nuclear protein 10 (NOP10), ceramide kinase (CERK), transmembrane protease, serine 13 (TMPRSS13), tripartite motif containing 37 (TRIM37), MEF2-interacting transcriptional repressor (MITR), synaptotagmin-like 4 (SYTL4), nod-like receptor protein 3 (NLRP3), single genes (protocadherin 17 gene (*PCDH17*) and jumonji and AT-rich interaction domain containing 2 (*JARID2*)), or clusters of genes. Clinical trials that address the interaction between biomarkers and treatment approaches are necessary to tailor therapy in TNBC [120].

#### 4.3.1. Biomarkers Predicting Resistance to Platinum-Based Therapy

Lnc DLX6-AS1, miR-105, miR-93-3p, 321 miRNAs including miR-34a, BRCAness, and HRD were reported to account for resistance to platinum-based therapy.

##### Lnc DLX6-AS1, miR-105, miR-93-3p and 321 miRNAs including miR-34a 

In a study, overexpression of DLX6-AS1 levels determined by quantitative real-time PCR (RT-qPCR) was found in TNBC tissues and cell lines when compared with that of normal tissues or breast fibroblast cells. Knockdown or upregulation of DLX6-AS1 decreased or increased cisplatin resistance, respectively. Moreover, findings in xenograft experiments using nude mice showed that DLX6-AS1 governed cell proliferation, EMT, and cisplatin resistance by miR-199b-5p/PXN axis [121]. MiR-105 and miR-93-3p induced cisplatin chemoresistance, stemness, and metastasis in TNBC through Wnt/beta-catenin signaling [122]. In a pilot study evaluating bloodborne miRNA signatures from 21 basal-like TNBC cases treated with NACT 321 deregulated miRNAs including miR-34a were reported when comparing expression pre- and post-treatment. Besides after NACT containing paclitaxel and carboplatin, the complete responders had a tendency to have higher miRNA levels [123].

##### BRCAness and HRD

*BRCA1/2* genes code for tumor suppressor proteins involved in DNA repair via homologous recombination therefore they play a critical role in genetic integrity. In particular, *BRCA* mutations lead to HRD and many patients were reported to harbor HRD [124]. However, HRD can occur in tumors that do not carry *BRCA1/2* mutation, defining a subgroup of patients referred to as BRCAness. BRCAness includes a series of traits in which *BRCA1* dysfunction following gene mutation, methylation, or deletion accounts for DNA repair deficiency [125]. BRCAness refers to a phenotype common in TNBC that shares molecular characteristics, and the resulting clinical features are similar to those found in *BRCA*-mutated patients [126]. Patients with a BRCAness phenotype have DNA repair failure and different mechanisms, including epigenetic inactivation of *BRCA* and germline or somatic mutations in other key genes involved in the homologous recombination system such as *BARD1*, *ATR*, *PALB2*, *RAD51*, *RAD51D*, *ATM*, *CHK1*, *PLK1*, and *WEE1* are responsible [126,127]. An HRD score was elaborated as a tool to further identify TNBC tumors that encompass a BRCAness phenotype occurring in roughly 45–70% of TNBC [128]. High HRD score is significantly associated with improved pCR rate with standard NACT in TNBC [129]. Identifying which TNBC tumors have HRD may further define the patients that would benefit from treatment with platinum agents [130]. In two clinical studies the HRD score predicted the likelihood of response to platinum-containing therapy in the neoadjuvant setting [131,132] and tumors with BRCAness may show similar sensitivities to anticancer drugs as tumors with *BRCA1* mutations. Another study investigated the association of *BRCA* mutations or BRCAness with drug sensitivities in TNBC. Namely, BRCAness as BRCA1-like score was evaluated in 12 TNBC cell lines, including four with mutations, using multiplex ligation-dependent probe amplification. Sensitivities to docetaxel, cisplatin, and epirubicin were compared with *BRCA* mutations and BRCA1-like scores. Sensitivity to cisplatin was examined in *BRCA1* knockdown MCF-7 cell lines. Eight- and four-cell lines had characteristics of BRCAness and non-BRCAness, respectively. Regarding cisplatin, scores were lower in *BRCA* mutants and tumors with BRCAness than their counterparts. An inverse correlation was found between BRCA1-like scores and cisplatin sensitivity (r = −0.407; *p* = 0.013) and *BRCA1* gene knockdown increased the cisplatin sensitivity of Michigan Cancer Foundation-7 cells. Authors concluded that BRCA1-like scores were associated with cisplatin sensitivity [133].

#### 4.3.2. Biomarkers Predicting Resistance to Taxanes Alone or with Other Agents

BRCAness, *IL-6, CXCL8, VEGFA*, early growth response 1 (*EGR1*), prostaglandin-endoperoxide synthase 2 (*PTGS2*), and tribbles pseudokinase 1 (*TRIB1*) signature, CXCL8-CXCR1/2 axis as well as SYTL4, MITR, serine protease inhibitor clade E member 1 (SERPINE1), tumor necrosis factor ligand superfamily member 13 (TNFS13), miR-5195-3p, miR18a, miR-1207-5p, metastasis-associated lung adenocarcinoma transcript 1 (MALAT1), CERK, transmembrane protease serine 13 (TMPRSS13), PCDH17, and JARID2 factors were reported as predictive biomarkers of resistance to taxanes alone or with other agents.

##### BRCAness 

In a study, BRCAness was detected in 121 breast cancer patients. Forty-eight patients (39.7%) were identified as BRCAness positive. Tumors of BRCAness were more likely to be hormone receptors negative (95.8% vs. 50.7%, *p* < 0.001), nuclear grade III (76.1% vs. 48.4%, *p* = 0.001) and TNBC subtype (91.6% vs. 42.5%, *p* < 0.001). In NACT subgroup analysis, clinical response rate for taxane-based regimen was significantly lower in BRCAness patients (58.3% vs. 77.8%, *p* = 0.041). Authors concluded that BRCAness may suggest resistance to taxane-based CT [134]. Similarly, in the just above-mentioned study [133] the 50% inhibitory concentration of docetaxel was higher in *BRCA* mutant and BRCAness cell lines than their counterparts. BRCA1-like scores showed a weak positive correlation with docetaxel sensitivity (*r* = 0.377; *p* = 0.039). Authors concluded that BRCA1-like scores were associated with docetaxel resistance.

##### *IL-6, CXCL8, VEGFA, EGR1, PTGS2, TRIB1* Signature and CXCL8-CXCR1/2 Axis

The implication of paclitaxel in TNBC cell lines after a prolonged administration, and the altered gene expression pattern by microarray technology and validation by qRT-PCR of the resistance to therapy relevant genes were evaluated. Functional assays showed that paclitaxel exhibits antiproliferative activity on Hs578T/Pax and MDA-MB-231/Pax demonstrating the activation of cell death mechanisms. Important alterations at the transcriptomic and genomic levels were observed. Particularly, a common drug resistance signature (*IL-6, CXCL8, VEGFA, EGR1, PTGS2* and *TRIB1*) for both cell lines at 24 passages was discovered. Also, an important mutation (tumor protein 53, *TP53*) linked with drug response was identified [135].

Another study investigated the prediction value of CXCL8-CXCR1/2 axis for TNBC patients undergone NACT with weekly paclitaxel plus carboplatin. Correlations between variables and treatment response were studied. CXCL8 level was significantly upgraded after NAC in CXCR1/2+ patients and downgraded after NACT in CXCR1/2- patients. Higher pCR rate was more likely observed in patients with lower CXCL8 level at surgery (*p* = 0.004, HR 0.939, 95% CI 0.900–0.980). Authors concluded that although further confirmatory studies are needed, these findings suggest that CXCL8-CXCR1/2 might play an important role in tailoring and modifying the NACT strategy for advanced TNBCs [136].

##### SYTL4, MITR, SERPINE1, TNFS13 Factors and miR-5195-3p, miR-18a, and miR-1207-5p, MALAT1, CERK, TMPRSS13, *PCDH17, JARID2*

In a study [137], it was hypothesized that the molecular profiling of tumor samples before and after NACT would be helpful in identifying genes likely responsible for drug resistance. Ten tissue samples were taken and sequenced by RNA-seq from eight patients with TNBC who underwent NACT. Three patients did and five patients did not have pCR. *SYTL4*, a Rab effector in vesicle transport, was considered a leading functional candidate. In particular, *SYTL4* in taxane-treated TNBCs was found being a novel chemoresistant gene as validated in TNBC cells, a mouse model and patient-derived organoids. Mechanistically, SYTL4 directly binds microtubules and decreases microtubule stability. In another investigational research [138] a genome-wide CRISPR screening combined with trancriptome analyses, was performed to identify candidates involved in paclitaxel-resistant TNBCs. Cell proliferation, cytotoxicity, immunofluorescent staining, and xenograft assays were carried out to verify the phenotypes of paclitaxel resistance induced by candidate genes, both in vitro and in vivo. MITR, the truncated isoform of histone deacetylase 9 (HDAC9) lacking the deacetylation domain, was enriched in paclitaxel-resistant cells. MITR overexpression resulted in IL11 hyper-expression and activation of downstream JAK/STAT3 signaling. Mechanistically, MITR counteracted MEF2A-induced transcriptional suppression of IL11, ultimately causing paclitaxel resistance. By contrast, pharmacological inhibition of JAK1/2 by ruxolitinib reversed paclitaxel resistance both in vitro and in vivo. Authors concluded that their study elucidated the principal role of MITR/MEF2A/IL11 axis in paclitaxel resistance so appointing a novel therapeutic strategy to improve responses to paclitaxel in TNBC patients. In an experimental study in TNBC cells [139] the role and mechanism of serine protease inhibitor, clade E member 1 (SERPINE1) were evaluated with reference to paclitaxel (PTX) resistance. A bioinformatic analysis of gene expression profiles in PTX resistant cells showed that *SERPINE1* was significantly associated with PTX resistance. Accordingly, SERPINE1 mRNA and protein levels were increased in PTX-resistant cells compared with those in PTX-sensitive parent cells. *SERPINE1* knockdown significantly inhibited cell survival and promoted cell apoptosis in vitro. In addition, *SERPINE1* silencing downregulated the key angiogenetic VEGFA. This study proved the oncogenic role of *SERPINE1* in PTX drug resistance of breast cancer and appointed it as a possible target for treating BC. In another study [140], endogenous expression of TNFSF13 in a panel of TNBC cell lines showed strong correlation with PTX and doxorubicin IC50 concentrations. While knocking down *TNFSF13* increases PTX efficacy in PTX-insensitive MDA-MB231 cells, recombinant *TNFSF13* (recTNFSF13) desensitizes PTX-sensitive HCC1806 cells to PTX. By in-silico analysis and western blotting, TNFSF13 expression was found to inversely correlate with the activity of the Akt-mTOR pathway, which acts as a negative regulator of autophagy activity. Consistent with this finding, the pharmaceutical inhibition of autophagy activity significantly re-establishes the efficacy of PTX in TNFSF13-treated HCC1806 cells. These findings suggest that *TNFSF13* promotes chemoresistance in TNBCs through autophagy initiation and that TNFSF13 overexpression accounts for a poor response to CT in TNBCs. 

Further studies revealed that the upregulation of miR-5195-3p, miR-18a, and miR-1207-5p is a potential predictor of TNBC sensitivity to paclitaxel [141,142,143]. 

MALAT1 is a highly conserved lncRNA, and it was found to be a potential biomarker in TNBC, helping to predict clinical outcome and resistance to neoadjuvant paclitaxel and doxorubicin [144].

CERK is a lipid kinase that plays a key role in the level of ceramide and ceramide 1-phosphate (C1P) by phosphorylating ceramide to produce C1P [145]. Ceramide induces apoptosis and is antiproliferative in many tumor cell types; conversely, C1P gives opposite effects [146,147,148]. It was reported that CERK overexpression strongly affects chemosensitivity and, regarding chemoresistance, can be a biomarker for risk stratification of newly diagnosed TNBC patients. Accordingly, CERK overexpression showed to be a biomarker for chemotherapeutic response in TNBC and higher than two-fold change in CERK (from tumor)/CERK (from normal counterpart) ratio was significantly linked to chemoresistance to doxorubicin and paclitaxel (OR = 2.66, 95% CI 1.18–7.34), *p* = 0.04. *CERK* overexpression conferred chemoresistance in TNBC cell lines that CERK inhibition allowed to overcome; mechanistic studies suggest that CERK mediates intrinsic resistance and lower response to CT in TNBC by governing several oncogenic pathways such as Ras (rat sarcoma virus)/ERK (extracellular signal-regulated kinase), PI3K/Akt/mTOR, and Ras homolog family member A (RhoA) [149].

The type II transmembrane serine proteases (TTSPs) are a family of cell-surface proteases that play critical roles in different cancers. In a study [150], systematic in silico data analysis, followed by immune-histochemical validation, identified increased expression of the transmembrane protease, serine 13 (TMPRSS13), in invasive ductal carcinoma tissue samples compared to that of normal breast tissue. Targeting TMPRSS13 expression renders aggressive TNBC cell lines highly responsive to paclitaxel and carboplatin. At the molecular level, knockdown of *TMPRSS13* in breast cancer cells led to increased protein levels of the tumor-suppressive protease prostasin, which was identified as a potential novel target for *TMPRSS13*. Regulation of prostasin levels may be a mechanism that contributes to the pro-oncogenic properties of *TMPRSS13* in breast cancer.

A study [151] investigated whether *PCDH17* gene methylation in TNBC tissues correlated with the effectiveness of NACT. Two-hundred-and-eighty TNBC patients were recruited, and diagnosis was made by core needle biopsy. Overall, 228 patients were positive for *PCDH17* methylation, and the 52 remaining were negative. Moreover, 107 patients had pCR after NACT. The pCR rate was 67.3% among the 52 patients negative for *PCDH17* methylation and 31.6% among the 228 patients positive for *PCDH17* methylation. Patients who were negative for *PCDH17* methylation and had high Ki67 expression showed significantly higher pCR rates than their counterparts. These findings suggest that *PCDH17* methylation status may predict the response to NAC in patients with TNBC. Another study enrolled 14 TNBC patients without pCR following NACT. In seven of them disease progressed within 12 months after mastectomy. Next generation sequencing (NGS) analysis targeting 422 cancer-related genes and in vitro studies was carried out. Among 422 cancer-related genes, alterations in 30 genes were found. *TP53* (12/14, 85.7%) was the most common mutated gene, while *RB1* mutations significantly occurred in patients with high Ki-67 scores (*p* = 0.013). Additionally, four mutations of *PTPN13* (57.1%, 4/7) and three of *JARID2* (42.9%, 3/7) were only observed in the short-DFS group, while patients with *JARID2* mutation had a significantly shorter DFS period (*p* = 0.026). After knockdown of *JARID2* in MD-MBA-231 cells by small interfering RNA (siRNA) the expression of E-cadherin reduced, and the levels of vimentin, MMP7, and MMP9 increased. Authors concluded that *JARID2* mutation and high tumor mutational burden (TMB) are potential prognostic and predictive biomarkers in TNBC patients [152].

#### 4.3.3. Biomarkers Predicting Resistance to Anthracyclines 

Circular RNAs (CircRNAs), miR-449 family, miR-770, a cluster of miRNAs, a cluster of genes, TNFSF13, and plasmacytoma variant translocation 1 (PVT1) factors were described as predictive biomarkers of resistance to anthracyclines.

CircRNAs are strongly involved in the initiation and progression of human cancers. A study [153] investigated mechanisms and the related functions of circUBE2D2 (hsa_circ_0005728) that account for TNBC progression and chemoresistance. The expression of circUBE2D2, miR-512-3p, and cell division cycle associated 3 (CDCA3) mRNA were assessed by qRT-PCR. Silencing of circUBE2D2 decreased doxorubicin resistance of TNBC cells. In-depth mechanism analysis uncovered that circUBE2D2 acted as a miRNA sponge to shield CDCA3 from the attack of miR-512-3p. Moreover, circUBE2D2 depletion induced tumor-suppression, which was importantly impaired upon miR512-3p downregulation or CDCA3 upregulation. Additionally, circUBE2D2 depletion diminished the resistance to doxorubicin through affecting miR-512-3p/CDCA3 axis. miRNA-449 family was found to mediate doxorubicin resistance in TNBC cells by governing cell cycle factors [154].

Abnormal expression of miR-770 can inhibit the resistance of TNBC cells to doxorubicin, mainly through regulation of apoptosis and TME [155]. The up/downregulation of an entire cluster of microRNAs, in particular, miR-221/222 and miR-200 families, was found to influence doxorubicin resistance in TNBC [156]. In a study, the effect of doxorubicin in TNBC cell lines was investigated and molecular alterations after a long exposure to doxorubicin were highlighted. In TNBC cell lines, doxorubicin exposure significantly increased the half maximal inhibitory concentration (IC50) values at P12 and P24 compared to that of parenteral cells P0; a total of 196 upregulated and 115 downregulated genes were observed as effects of multiple dose exposure, and 15 overexpressed genes were found to be involved in drug resistance. Also, the presence of some additional mutations in both cell lines was observed. The outcomes of this research may provide novel biomarkers for drug resistance in TNBC. Also, this activity can highlight the potential mechanisms associated with drug resistance, as well as the potential therapies to counteract these mechanisms [157]. In [140], immunohistochemistry findings showed that TNFSF13 protein overexpression occurred in TNBC patients not responding to an anthracycline-based therapy. In a further study [158], plasmacytoma variant translocation 1 (*PVT1*) increased the resistance of the TNBC cell line MDA-MB-231 to doxorubicin. It was found that PVT1 promoted the protein stability of nuclear factor erythroid 2 like 2 (Nrf2) by inhibiting the binding of kelch-like ECH-associated protein 1 (Keap1) to Nrf2. This induced the resistance of MDA-MB-231 cells to doxorubicin. In another study [159], it was reported that the TRIM37 network affects TNBC tumors allowing tumor cells to resist doxorubicin. Particularly, it was found that TRIM37-directed histone H2A monoubiquitination increased changes in DNA repair that made *TP53*-mutant TNBC cells resistant to CT. Besides, chemotherapeutic drugs promoted a positive feedback loop via ATM/E2F1 (E2F transcription factor 1)/STAT signaling that boosted the TRIM37 network in chemoresistant cancer cells. 

#### 4.3.4. Biomarkers Predicting Resistance to Gemcitabine and CMF

Gemcitabine is often used in the treatment of breast cancer and NLRP3 and mir-620 were reported to predict resistance to this drug. In a study conducted in TNBC cells and gemcitabine-resistant cell (GRC) lines [160] the potential mechanism of gemcitabine resistance was investigated. In particular, the sensitivity to different concentrations of gemcitabine with reference to regulation of Nod-like receptor protein 3 (NLRP3) expression was assessed. NLRP3 mRNA expression was determined by RT-PCR and MTT assay evaluated the cell cytotoxicity. NLRP3 overexpression prolonged cell survival and decreased sensitivity to gemcitabine (*p* < 0.05). NLRP3 was highly and more expressed in GRC than in TNBC cells. GRC viability strongly decreased as the gemcitabine concentration increased and NLRP3 overexpression enhanced resistance to gemcitabine in GRC (*p* < 0.05). NLRP3 agonists might induce EMT, promote wnt/beta-catenin signaling and IL-1β, while switching off wnt/beta-catenin signaling could result in the inhibition of NLRP3, IL-1β and EMT as well as cell viability in GRC (*p* < 0.05). Overall, this suggests that NLRP3 increases resistance to gemcitabine through IL-1-beta/EMT/Wnt/beta-catenin pathway. miR-620 upregulation in TNBC cells promotes gemcitabine resistance by reducing deoxycytidine monophosphate deaminase (DCTD) expression [161]. NOP10, which is involved in ribosome biogenesis and telomere maintenance, also plays a crucial role in carcinogenesis. In a study [162] NOP10 mRNA levels were investigated using the Molecular Taxonomy of Breast Cancer International Consortium (METABRIC) (*n* = 1980) and Cancer Genome Atlas (TCGA) BC cohorts (*n* = 854). In CT-treated patients (CMF regimen), NOP10 protein overexpression, independent of tumor size and grade and nodal stage, was significantly associated with shorter survival (*p* = 0.03), higher risk of death (*p* = 0.028) and occurrence of distant metastasis (*p* = 0.02). Authors conclude that NOP10 expression can predict CT resistance and that “functional assessments are necessary to decipher the underlying mechanisms and to reveal its potential therapeutic values in various BC subtypes especially in the aggressive TNBC class”. 

The main biomarkers predictive of response or chemoresistance and their characteristics are reported in Table 2A,B.

## 5. Drugs Currently Recommended or Helpful in Chemoresistant TNBC

Recently, based on specific biomarkers, some therapies were tailored to TNBC subsets and became available in clinical practice: olaparib and talazoparib for *BRCA1/2* germline mutations carriers; larotrectinib and entrectinib for *NTRK* gene fusion carriers; anti-trophoblast cell surface antigen 2 (Trop2) antibody drug conjugate therapy. Other targeted therapies are under investigation [163].

### 5.1. Polymerase ADP-Ribose Inhibitors (PARPi) Are Recommended in TNBC BRCA1/2 Germline Mutation Carriers 

About 10–20% of TNBC has *BRCA1/2* germline (g*BRCA*) mutation. In the POSH study, no significant difference in OS was found in positive versus negative g*BRCA* carriers. Despite this, when the primary analysis in patients with TNBC excluding 37 (7%) patients who developed a new primary breast or ovarian cancer was repeated, OS at 10 years was 78% (95% CI 69–85) in *BRCA*-positive versus 69% (64–74; HR 1.24 [95% CI 0.39–3.96], *p* = 0.73 in *BRCA*-negative patients [164]. Better response to conventional CT depending on HRD deficiency or better immune response might explain the prolonged OS in g*BRCA* TNBC. Particularly, in the former instance the activity of DNA-damaging agents such as platinum salts and PARPi should be increased by g*BRCA 1/2* mutations. In two Phase III trials carried out in metastatic setting, significantly increased PFS by PARPi as monotherapy compared with standard CT occurred in patients with g*BRCA1*/*2* mutated breast cancer [165,166]. Currently, olaparib is recommended in adjuvant and metastatic settings, while talazoparib is recommended in metastatic setting alone of g*BRCA* carriers [12,13]. In the Phase II PETREMAC trial, patients with primary TNBC more than 20 mm received neoadjuvant olaparib for up to 10 weeks before CT. Eighteen out of thirty-two patients showed an objective response (OR) to olaparib (56.3%). Sixteen out of eighteen responders compared to 4/14 nonresponders, had homologous recombination (HR) mutations and/or *BRCA1* methylation [167]. In a study, 107 patients with untreated primary HER2-negative and TNBC with HRD were randomized either to paclitaxel plus olaparib for 12 weeks or paclitaxel plus carboplatinum for 12 weeks, both followed by epirubicin/cyclophosphamide (EC). The pCR rate with paclitaxel-olaparib was 55.1% versus paclitaxel-carboplatin 48.6% [168]. In another neoadjuvant study, 20 patients with HER2 negative, g*BRCA*-positive disease received six months of once per day oral talazoparib, followed by definitive surgery; fifteen patients had TNBC; pCR rate was 53% [169]. PARPi are under evaluation in combination with CT in neoadjuvant setting (NCT03740893, NCT03150576, NCT02789332), or in combination with chemo and/or immunotherapy in advanced TNBC (NCT03801369, NCT02484404, NCT04690855).

### 5.2. Larotrectinib and Entrectinib for NTRK Gene Fusion Carriers

About 1% of all solid tumors show somatic chromosomal rearrangements involving the neurotrophic tropomyosin receptor kinase (*NTRK1*, *NTRK2,* and *NTRK3*) genes [170]. Tumor growth promotion derives from TRK gene fusion through overexpression of the proteins and their constitutive downstream activation. The efficacy of larotrectinib, a tropomysin receptor kinase inhibitor, was assessed in the LOXO-101 trial, which showed 71% OR rate and led to FDA approval [170,171]. Entrectinib, another tropomysin receptor kinase inhibitor that proved to be efficacious for patients with *NTRK*-fusion-positive solid tumors [172], was successively approved by the FDA. *NTRK* fusions occur, more similarly than in other types, in less than 1% breast cancers. Ross et al., using comprehensive genomic profiling, identified only 16 tumors (0.13%) with *NTRK* gene fusions among 12,214 locally aggressive, relapsed, or metastatic breast cancers. Among them, nine cases were ductal carcinomas, and three were secretory carcinomas. All tumors were HER2-negative, more often TNBC, and the majority had *NTRK1* fusions [173]. Interestingly, human secretory breast carcinoma is less than 0.02% of all breast cancers [174], and very often (above 90%) harbor ETS variant transcription factor 6 (*ETV6)-NTRK3* gene fusion previously cloned in pediatric mesenchymal cancers [175]. Most secretory breast carcinoma are classified by genomic profiling as basal-like tumors with triple-negative receptor status [176,177]. However, *ETV6-NTRK3* gene fusion is often associated with indolent, slow-growing tumors. This highlights the molecular heterogeneity of TNBCs [178]. To date, in 15 patients with metastatic breast cancer treated with these tropomysin receptor kinase inhibitors, response rates of approximately 80% were reported [171,172,179]. Metastatic breast cancer harboring *NTRK* fusions and progressing despite previous treatment is approved for receiving TRK inhibitors [12,13].

### 5.3. Anti-Trop2 Antibody Drug Conjugate Therapy

Trop-2 is a glycoprotein overexpressed in multiple epithelial cancers that accounts for pro-growth signaling [180]. Sacituzumab govitecan-hziy is an anti-Trop-2 antibody conjugated to an active metabolite of irinotecan (SN-38) [181,182]. This drug inhibits topoisomerase activity and its DNA binding, impedes ligation of cleaved DNA strands and gives rise to double-strand DNA breaks, induces cell death, and blocks DNA replication in tumor cells [180,181]. In heavily pretreated mTNBC patients [182,183,184], sacituzumab govitecan-hziy improved response rate and median PFS compared to that of standard CT (33.3% and 5.5 months vs. 10–15% and 2–3 months respectively) [184]. The phase 3 ASCENT trial (NCT02574455), a randomized study carried out in the same type of patients to validate the safety and efficacy data [185] was stopped due to the evidence of drug efficacy. The mTNBC patients receiving sacituzumab govitecan-hziy had a PFS of 5.6 months (95% CI, 4.3–6.3), compared to 1.7 months for patients who received CTs of physician’s choice (*p* < 0.0001) [184]. In 2020, Sacituzumab govitecan-hziy received accelerated FDA approval for heavily pretreated and advanced mTNBC.

### 5.4. Other Emerging Targeted Therapies 

#### 5.4.1. Targeting Pathological TGF-Beta, Notch, Wnt/Beta-Catenin, Hedgehog, NF-kB, the PI3K-AKT-mTOR, and STAT3/JAK Molecular Pathways

CT-induced TGF-β signaling enhances tumor recurrence through IL-8-dependent expansion of CSCs and TGF-β pathway inhibitors prevent the development of drug-resistant CSCs. Thus, a combination of TGF-β inhibitors and anticancer CT could be useful in patients with TNBC [24]. An ongoing Phase I clinical trial is investigating galunisertib, a potent inhibitor of TGF beta type I receptor, in combination with CT in metastatic TNBC (NCT02672475). 

In breast cancer cell lines, doxorubicin induced Notch-1 signaling which led to increased ABCC1 expression. Gamma-secretase inhibitor (GSI) inhibited the Notch-1 upregulation of ABCC1, thus rendering the cells more susceptible to doxorubicin [71]. This effect was confirmed in TNBC cells, where GSI enhanced the efficacy of doxorubicin [63]; GSIs-CT combination to treat advanced breast cancer, including TNBC, was investigated in two phase I clinical trials. PF-03084014 GSI, combined with docetaxel, was well tolerated and showed clinical benefit in patients with advanced TNBC [186]. In a recent preclinical study conducted in TNBC patient-derived xenografts with abnormal Notch signaling, a novel GSI, AL101, showed important antitumor effects [185].

Wnt/beta-catenin inhibitors, such as SRI33576, SRI35889, and salinomycin, can inhibit breast CSC proliferation, invasion, and self-renewal in addition to induce apoptosis [187,188]. CWP232228, which inhibits Wnt pathway signaling by blocking nuclear beta-catenin interaction with T-cell factor, decreased tumor growth in TNBC xenograft models and was strongly efficacious against chemoresistant breast CSC both in vitro and in vivo [54]. A repurposed drug, clofazimine, decreased the proliferation of TNBC cells and tumor growth in xenograft models. Moreover, clofazimine showed a relevant synergistic effect with doxorubicin with a good tolerability [189]. A recombinant human Frizzled-7 protein antagonist (rhFzd7) decreased proliferation, invasion, and angiogenesis by inhibiting Wnt/beta-catenin pathway, while sensitizing TNBC cells to docetaxel both in vivo and in vitro [190]. LGK974, a small molecule blocking Wnt ligand secretion, is under evaluation in patients with Wnt-ligand dependent malignancies, including TNBC (NCT01351103). Similarly, PTK7-ADC, an antibody–drug conjugate targeting a component of the Wnt/beta pathway, is currently assessed as a therapeutic combination in metastatic TNBC (NCT03243331). 

The majority of Hh signaling pathway inhibitors are directed against SMO. However, their efficacy in breast cancer, including TNBC, was disappointing. SMO independent activation of the Hh pathway was demonstrated in TNBC and could partially account for the lack of efficacy of SMO inhibitors [191]. Preclinical data indicate that the use of GLI inhibitors might be preferred for TNBC treatment. GANT61, a direct GLI inhibitor, promoted apoptosis, decreased proliferation, and CSC population in TNBC cell lines [192,193]. However, so far, none of the GLI inhibitors were entered into clinical trials.

Most NF-kB inhibitors are nonspecific as they affect many other targets besides the NF-kB pathway. This and the pleiotropic effects of NF-B likely account for their high toxicity [194]. Plumbagin, a nonspecific inhibitor, and genistein, a major soy isoflavone inhibiting NF-kB activity via Notch-1 pathway, exert anti-growth and pro-apoptotic effects in TNBC cells [195,196]. Dehydroxymethylepoxyquinomicin (DHMEQ), which inhibits nuclear translocation of NF-B, decreased growth and induced apoptosis in TNBC cells, likely by reducing the activation of this pathway [197].

Targeting the PI3K-AKT-mTOR pathway together with CT can be a useful strategy in aggressive TNBCs with *PTEN* loss. Everolimus, an mTOR inhibitor, was effective against TNBC in preclinical investigations. Promising results were also obtained for NVP-BEZ235, a PI3K/mTOR inhibitor, in TNBC cell lines [102] and several Phase I and II clinical trials investigating the effects of mTOR and PI3KA inhibitors, alone or in combination with CT, mainly in advanced TNBC are underway (NCT02531932, NCT01931163, NCT01629615, NCT04216472). Recently, AKT proved an important therapeutic target in advanced/metastatic TNBC. A combination of the AKT inhibitor ipatasertib with paclitaxel prolonged PFS and OS of TNBC patients compared to paclitaxel alone. A greater benefit occurred in patients with alterations in the molecular PIK3CA/AKT1/PTEN pathway thus highlighting the relevance of careful patient selection [198]. Accordingly, an ongoing trial is investigating ipatasertib in advanced TNBCs preselected for PIK3CA/AKT1/PTEN alterations (NCT03337724). Uprosertib, another AKT inhibitor, is under evaluation in a Phase II clinical trial on metastatic TNBC (NCT01964924). AZD5363, a novel AKT inhibitor evaluated combined with CT in metastatic TNBC, prolonged OS in a Phase II trial [199].

Promising preclinical results targeting STAT3 and JAK2 in solid tumors including breast cancer were followed by a few clinical studies [200]. For example, JAK1/2 inhibitor ruxolitinib in combination with NACT and AZD9150, a novel antisense nucleotide inhibitor of *STAT3*, together with durvalumab and paclitaxel are under investigation in triple-negative inflammatory breast cancer (NCT02876302) and in a Phase I/II clinical trial in metastatic TNBC (NCT03742102), respectively.

#### 5.4.2. Targeting Apoptosis, miRNAs, EGFR, and AR

Many studies among anticancer strategies focused on Bcl2 family members, TRAIL receptors, and inhibitors of apoptosis (IAPs) [201]. A recent phase II clinical study conducted in metastatic TNBC and investigating tigatuzumab combined with CT was unsuccessful [202]. MEDI3039, a novel death receptor (DR) multivalent agonist, showed elevated antitumoral efficacy both in-vitro and in-murine models of TNBC [203]. Following pro-apoptotic stimuli, mitochondria release the second mitochondria-derived activator of caspases (SMAC) which acts as an antagonist of IAPs. Thus, SMAC mimetics were constructed as proapoptotic, anticancer agents that could be particularly effective in TNBC [204]. For example, Debio 1143 (AT406) with good preclinical results is under investigation in several Phase I trials on advanced solid tumors, including TNBC (NCT01078649, NCT01930292). In preclinical studies, another SMAC mimetic, LCL161 promoted apoptosis and showed synergistic effects with paclitaxel. Particularly, in a phase II clinical trial, LCL161 administered as a neoadjuvant agent in association with paclitaxel was highly effective; in fact, in localized TNBC, LCL161/paclitaxel combination more than doubled the pCR rate compared with that of paclitaxel alone, although with increased toxicity. However, the pCR effect was only present in the TNBC group preselected for the tumor necrosis factor (*TNF*) gene expression profile [205].

Regarding therapeutic involvement of miRNAs, two basic strategies were developed: oncogenic miRNAs inhibition and the use of substitutes for rehabilitation of tumor suppressor miRNAs function [206]. Anti-miRNA oligonucleotides, miRNA sponges, small RNA zipper molecules, antagomiRNAs, locked nucleic acid anti-miRNAs, and small molecule inhibitors are the agents commonly used to inhibit oncogenic miRNAs. Antisense-miRNAs and restoration of tumor suppressor miRNAs using miR-mimics inhibited TNBC growth, migration, and invasion in cell lines and xenograft models [207,208]. MiRNAs-based therapeutic approach seems promising, although further improvements in delivery systems, toxicity, selectivity, and specificity are needed. 

*EGFR* activation/amplification was detected in approximately 25–50% of TNBC [209,210], and therefore EGFR inhibition should be effective in the treatment of EGFR-driven TNBC. In TNBC, mAbs specific for the receptor and the use of tyrosine kinase inhibitors (TKIs) are two common strategies used for targeting EGFR (and other receptor tyrosine kinases). Cetuximab, an anti-EGFR mAb evaluated in metastatic TNBC in association with cisplatin, moderately increased PFS and OS [211]. However, a Phase II study of cetuximab in combination with carboplatin in metastatic TNBC obtained disappointing results [212]. Panitumumab, another EGFR mAb, showed different efficacy in clinical trials [213,214] and clinical trials of panitumumab in combination with CT in inflammatory TNBC are ongoing (NCT02876107, NCT01036087). Among TKIs, promising findings were reported for apatinib in TNBC [86,215,216,217]. A clinical trial investigating icotinib in metastatic TNBC is currently recruiting patients (NCT02362230), while the association of anti-EGFR mAbs and TKIs could result in a stronger antitumor action likely due to a synergistic effect [218]. However, in TNBC, although it is a tumor characterized by relatively high rate of EGFR overexpression EGFR, targeted therapy has poor performance. The “EGFR paradox” could explain this phenomenon. According to this hypothesis, EGFR signaling changes during tumor progression, and while EGFR is overexpressed in primary tumors, metastatic cells become intrinsically resistant to EGFR targeted therapy. Accordingly, the two clinical studies of panitumumab that reported the greatest benefit were conducted on operable, primary TNBC [213,219].

Findings from clinical and preclinical studies suggest that LAR is a resistant subtype [104]. LAR tumors are relatively quiescent, which at least in part could explain their CT resistance [106]. Bicalutamide, a first-generation AR antagonist, induces cell apoptosis and inhibits cell motility and invasiveness in cell line MDA-MB-453 [220] and cell lines representing the LAR subtype are sensitive to AR antagonist bicalutamide and 17-DMAG [102]. In a first phase II study of metastatic AR-positive TNBC breast cancer patients treated with bicalutamide, a six-month clinical benefit rate of 19% and a median PFS of 12 weeks occurred [221]. In another Phase II single-arm trial conducted in 146 AR-positive TNBC patients with inoperable locally advanced or metastatic diseases whose tumors had > 10% AR expression, a different AR inhibitor, abiraterone acetate plus prednisone, showed comparable results to bicalutamide [222]. Enzalutamide, a second-generation AR antagonist, showed clinical activity in a Phase II study recruiting patients with locally advanced or metastatic AR-positive TNBC [223]. Moreover, AR inhibition with enzalutamide was an inductor of radiation sensitivity in AR-positive TNBC cell lines, proposing AR inhibition as a radio-sensitization strategy [224]. The START trial (NCT03383679) is an ongoing randomized Phase II study testing the efficacy of darolutamide, a new AR antagonist, compared to capecitabine for AR-positive, locally recurrent, or metastatic TNBC.

Drugs currently recommended or potentially helpful in chemoresistant TNBC are reported in Table 3A–C.

## 6. Discussion and Conclusions

Among breast cancer subtypes, TNBC is associated with the worst prognosis [2,4], and in spite of efforts performed in the last decades, no significant improvement in PFS and OS was obtained [225,226]. At present, CT is the mainstay treatment in TNBC; however, resistance to CT frequently occurs. However, TNBC is a heterogeneous disease, and many molecular mechanisms are involved in chemoresistance. Identification of these mechanisms is of particular relevance, as it can help in improving prognosis and therapy. Some biomarkers capable of predict resistance to specific chemotherapeutic agents were identified and are expected to be validated in future studies. These predictive factors could guide the therapeutic approach in both early and advanced disease. Current guidelines recommend NACT in operable TNBC > 2 cm or for breast conservation or in cN+ disease likely to become cN0; recently, NACT was considered not an option, but rather the preferred treatment strategy for TNBC patients in clinical practice [15]. However, disease progression during NACT is a potential risk [12,13]. Therefore, both in neoadjuvant and advanced settings, a more tailored approach and a more accurate selection of the employed drugs are main aims. Many studies based upon molecular biology defined the use of new drugs that could be essential in identifying the mechanisms accounting for chemoresistance to a specific antiblastic in each patient. Therefore, emerging therapies allow to select specific antiblastics that, alone or by integrating the conventional therapeutic approach, may overcome/hinder chemoresistance. 

In particular, PARP inhibitors improved prognosis in metastatic *BRCA* mutated patients [165,166] and are under evaluation in the neoadjuvant setting; TRK inhibitors showed activity and are approved in rare metastatic breast cancers harboring *NTRK* fusions and progressing despite previous treatment [171,172]; sacituzumab govitecan, based on the results of the phase III ASCENT trial, showed a PFS of 5.6 months compared to 1.7 months for patients who received chemotherapies of physician’s choice, and received accelerated FDA approval for pretreated and advanced metastatic TNBC [184]. However, some criticism arose around the results and the cost/effectiveness ratio of this trial [227,228]. PI3K/Akt/mTOR and EGFR inhibitors as well as antiandrogens showed promising results and are under evaluation in Phase II/III clinical trials. Immunotherapy is another interesting option. However, pembrolizumab or atezolizumab combined with CT increased the median PFS 4.1 and 2.5 months, respectively, and the clinical benefit was modest. Only about 40% of TNBCs are PD-L1 + and not all PD-L1 + patients with advanced TNBC respond to PD-L1 inhibitors. It is likely that redundant pathways of immune suppression are active in breast cancer or that important pathways of immune activation are silent. Therefore, new strategies targeting multiple pathways of immunoregulation [229] can improve the efficacy of the currently available and other new developed immunotherapies.

## Figures and Tables

**Table 1:** Principal reasons likely responsible for chemoresistance in triple-negative breast cancer (TNBC).

Biological Condition/ Component	Status	Mechanism	References
Cancer stem cells	Intrinsically enriched, increased after NACT through HIFs and ABC B1 upregulation	Quiescence, ABCG2 transporter overexpression, tumor-initiating cells enrichment	[17,18,19,20,21,22,23,24,25,26,27]
ABC Transporters	ABCC1/MRP1, ABCG2/BCRP, ABCC11/MRP8 intrinsic increase or after NACT or Hh pathway	Transporter-mediated efflux through ATP	[29,30,31,32,33]
Hypoxia	Morphological features characteristics of hypoxia (expression of CAIX)	Insufficient drug penetration and multiple other mechanisms due to the promoted TME changes (see text)	[34,35,36,37,38,39,40,41,42,43]
Apoptosis	Malfunction (BCL-2 and Mcl-1 protein expression)	Evasion of apoptosis	[44,45,46,47,48]
Factor			
EGFR	Increased expression (from 13% to 76%)	ABCG2-mediated, apoptosis inhibition, angiogenesis, and cell proliferation involvement	[6,49,50,51]
IGF-1R	Expressed in 46% and increased after NACT	ABCG2-mediated, apoptosis inhibition, angiogenesis and cell proliferation involvement, Wnt-beta-catenin interaction, CSCs self-renewal decrease	[52,53,54,55]
ADAM10	Highly expressed in cell lines	Notch signaling downregulation; proliferation, migration, invasion increase	[56]
NcRNAs	Aberrant expression	Promotion of apoptosis resistance, EMT, ABC transporters upregulation; cell cycle arrest, CSCs, DNA repair and autophagy inhibition	[57,58]
DNA methylation	Strong hypomethylation and low gains of methylation	Significantly differentially methylated regions	[59,60,61]
Phosphoproteome, phosphorylation of kinases	Activation of protein kinases and phosphatases through phosphorylation	Changes in phosphorylated proteins, phosphorylation and signal transduction involvement, aberrant expression or activation of protein kinases	[62]
Pathologic Molecular Pathway			
TGF-beta	Signaling increase after NACT	CSCs upregulation, EMT increase	[24,66,67,68,69]
Notch	Signaling increase after NACT	CSCs maintenance, ABCC1 overexpression	[70,71,72,73]
Wnt/beta-catenin	Signaling deregulation	CSCs increase, beta-catenin synergistic effect with NeK2B FLD8-mediated signaling increase	[74,75,76,77,78]
Hedgehog (Hh)	Signaling activation by cytotoxic drugs	CSCs expansion through GLI1/2 activation, promotion of expression of ABC transporters	[31,79,80,81,82]
NF-kB	Overexpression	Apoptosis inhibition	[83,84,85,86]
PTEN and PI3K-AKT-mTOR (PAM)	Hyperactivation due to PTEN loss	PTEN loss, HIF-1 induction by Akt	[87,88,89,90]
JAK/STAT	STAT3 hyperexpression downstream of IL-6/8 extracellular ligands	STAT3-NFkB interaction, STAT3 HIF-1 and ABC transporters expression upregulation	[91,92,93,94,95,96,97]

TNBC: triple negative breast cancer; CSCs: cancer stem cells; NACT: neoadjuvant chemotherapy; HIFs: hypoxia inducible factors; ABC: ATP binding cassette; MRP1: multidrug-resistant protein-1; BCRP: breast cancer resistance protein; CAIX: carbonic anhydrase IX; TME: tumor microenvironment; Bcl-2: B-cell lymphoma 2; Mcl-1: myeloid cell leukemia-1; EGFR: epidermal growth factor receptor; IGF1R: insulin growth factor 1 receptor; Wnt: wingless and Int 1; ADAM-10: a disintegrin and metalloproteinase-10; NcRNA: non coding RNA; TGF-beta: tumor growth factor-beta; EMT: epithelial-to-mesenchymal transition; Nek2B: NIMA-related kinase 2B; FZD8: frizzled-8; GLI: glioma-associated oncogene transcription factors; NFkB: nuclear factor kappa light chain enhancer of activated B cells; PTEN: phosphatase and tensin homolog; PI3K: phosphoinositol-3 kinase; Akt: akr mouse strain thymoma; mTOR: mammalian target of rapamycin; JAK: janus kinase; STAT: signal transducer and activator of transcription; IL: interleukin.

**Table 2:** Prediction of response or resistance (R) to chemotherapy (CT) in TNBC. B. Prediction of response or R to chemotherapy (CT) in TNBC.

A.					
Predictive Modality	Setting		Objective		References
	CS, ES	Kind	Outcome	Drug	
Histologic/Molecular subtype					
Metaplastic	CS	Neoadjuvant	Low pCR	Anthracycline/taxane-based NACT with or without carboplatin	[100]
LAR and MES				Carboplatin plus docetaxel	[104]
BL1 and BL2	ES	NA	High proliferation	Cisplatin	[102]
TILs					
Whole TILs	CS	Neoadjuvant	High pCR (positive correlation)	Anthracycline/taxane-based NACT	[107,108,109,110]
TILs, PD-L1, CD73 (TNP)					[111]
CD3+ cells					[112]
CD4+, CD8+, FOXP3+ cells					[113]
CD20+ cells					[105,107]
NK cells					[114,115]
Blood PMN neutrophils					[119]
Blood DCs					[119]
Biomarkers					
HE LncDLX6-AS1	ES	NA	R	Cisplatin	[121]
HE miR-105 and miR-93-3p					[122]
321 miRNAs (including miR-34a) expression change	CS	Neoadjuvant	High pCR	Carboplatin/paclitaxel	[125]
High HRD score				Anthracycline and/or taxane-based NACT	[129]
HRD				Platinum-containing NACT	[131,132]
Low BRCA1-like score	ES	NA	R	Cisplatin, docetaxel	[133]
BRCAness	CS	Neoadjuvant	Low pCR	Taxane-based NACT	[134]
*IL-6, CXCL8, VEGFA, EGR1, PTGS2, TRIB1* signature	ES	NA	R	Paclitaxel	[135]
LE CXCL8-CXCR1/2 axis	CS	Neoadjuvant	High pCR	Carboplatin plus paclitaxel	[136]
**B.**					
**Predictive Modality**	**Setting**		**Objective**		**References**
	**CS, ES**	**Kind**	**Outcome**	**Drug**	
Biomarkers					
HE SYTL4	CS/ES	Neoadjuvant/NA	R	Paclitaxel	[137]
HE MITR	ES	NA			[138]
HE SERPINE1					[139]
HE TNFS13				Paclitaxel, anthracycline	[140]
LE miR-5195-3p				Paclitaxel	[141]
HE miR-18a	CS	Neoadjuvant		Paclitaxel-containing NACT	[142]
HE miR-1207-5p	ES	NA		Paclitaxel	[143]
HE Long nc RNA MALAT-1	CS	Neoadjuvant		Paclitaxel/doxorubicin	[144]
HE CERK	CS/ES	Metastatic/NA			[149]
HE TMPRSS13	ES	NA		Paclitaxel/carboplatin	[150]
High *PCDH17* methylation	CS	Neoadjuvant		Taxane/anthracycline-based NACT	[151]
*JARID2* mutation			Short DFS in patients without pCR		[152]
circUBE2D2/miR-512-3p/CDCA3 axis	ES	NA	R	Doxorubicin	[153]
HE miRNA-449 family	CS	Neoadjuvant	S		[154]
HE miR-770	ES	NA			[155]
LE miR221/222 and miR200 family	CS	ND	R		[156]
A cluster of 15 overexpressed genes	ES	NA			[157]
HE PVT1					[158]
HE TRIM37 network					[159]
HE NLRP3				Gemcitabine	[160]
HE mir-620					[161]
NOP10	CS	Adjuvant	Short OS	CMF-treated	[162]

CS: clinical setting; ES: experimental setting; NA: not applicable; HE: high expression; R: resistance; LE: low expression; ND: not defined; S: sensitivity.

**Table 3:** Drugs currently recommended or potentially helpful in chemoresistant TNBC.

A.				
Drug	Target/Mechanism of Action	CS/ES	Outcome	Reference/NCT Number
Currently recommended				
Olaparib	PARP inhibitor	Metastatic, in HER2 negative BC pts with a germline *BRCA* mutation (CS)	Higher objective RR and PFS	[165]
Talazoparib		Advanced, in BC pts with germline *BRCA* mutation (CS)		[166]
Larotrectinib	Inhibitor of tropomyosin receptor kinase (TRK)	Advanced, in NTRK gene fusion-positive solid tumours (CS)	ORR 71%	[170]
Entrectinib			ORR 57%; Median duration of response 10 months	[172]
Sacituzumab govitecan	Anti-Trop2 antibody drug conjugate	Metastatic, in heavily pretreated pts (CS)	RR 33.3%; median duration of response 7.7 months; clinical benefit rate 45.4%; median PFS 5.5 months; OS 13.0 months	[184]
Under investigation				
Galunisertib	TGF beta type I receptor inhibitor	Metastatic, in combination with CT (CS)	NA	NCT02672475 (phase I)
PF-03084014	Gamma secretase inhibitor	Advanced, in combination with docetaxel (CS)	Median PFS 4.1 months	[186]
AL101		Patient-derived xenografts with abnormal Notch signaling (ES)	Inhibition of tumor growth	[185]
SRI33576, SRI35889	wnt/beta-catenin inhibitors	Cell lines (ES)	Pro-apoptotic effects by downregulating LRP6	[187]
Salinomycin		Breast CSCs (ES)	inhibition of proliferation, invasion, and self-renewal while inducing apoptosis	[185,186]
CWP232228		Xenograft models (ES)	Inhibition of tumor growth	[54]
Clofazimine		Cells and xenograft models (ES)	inhibition of proliferation;	[189]
Frizzled-7 protein antagonist (rhFzd7)		Cells and xenografts (ES)	Inhibition of proliferation, invasion, and angiogenesis while sensitizing cells to docetaxel	[190]
LGK974		Advanced, in pts with wnt-ligand dependent malignancies, including TNBC (CS)	NA	NCT01351103 (phase I)
PTK7-ADC		Metastatic, in combination with gedatolisib (dual PI3K-mTORC1/2 inhibitor) (CS)		NCT03243331 (phase I)
**B.**				
**Drug**	**Target/Mechanism of Action**	**CS/ES**	**Outcome**	**Reference/NCT Number**
Under investigation				
GANT61	Hh/direct GLI inhibitor	Cell lines (ES)	promoted apoptosis, reduced proliferation, and decreased CSC population	[192,193]
Plumbagin	Non-specific NF-kB inhibitor		Decreased cell viability and promoted apoptosis	[195]
Genistein	NF-kB inhibitor		Anti-growth and pro-apoptotic effects	[196]
DHMEQ	Nuclear translocation of NF-B inhibitor		Decreased growth and induction of apoptosis	[197]
Everolimus	mTOR inhibitor	Advanced, in combination with carboplatin (CS)	NA	NCT02531932 (phase II)
		Advanced, in combination with cisplatin (CS)		NCT01931163 (phase II)
BKM120	PI3K inhibitor	Metastatic (CS)		NCT01629615 (phase II)
Alpelisib		Neoadjuvant, in combination with nab-paclitaxel in anthracycline refractory pts with PIK3CA or PTEN alterations (CS)		NCT04216472 (phase I)
Ipatasertib	AKT inhibitor	Locally advanced/metastatic, first line (phase II), in combination with paclitaxel (CS)	Prolonged PFS and OS	[198]
Ipatasertib		Advanced, in PIK3CA/AKT1/PTEN-altered pts, in combination with paclitaxel versus placebo + paclitaxel (CS)	NA	NCT03337724 (phase III)
Uprosertib		Metastatic, in combination with trametinib (CS)		NCT01964924 (phase II)
AZD5363		Metastatic, in combination with CT (CS)	Prolonged OS	[201]
Ruxolitinib	JAK1/2 inhibitor	Neoadjuvant, in combination with CT (CS)	NA	NCT02876302 (phase II)
**C.**				
**Drug**	**Target/Mechanism of Action**	**CS/ES**	**Outcome**	**Reference/NCT Number**
Under investigation				
AZD9150	Antisense nucleotide inhibitor of *STAT3*	Metastatic, in combination with durvalumab and paclitaxel (CS)	NA	NCT03742102 (phase I/II)
MEDI3039	Apoptosis/DR agonist	In-vitro and in-murine models (ES)	Tumor growth inhibition	[205]
Debio 1143	IAP antagonist	Advanced, solid tumors including TNBC (CS)	NA	NCT01078649, NCT01930292 (phase I)
LCL161	SMAC analog	Neoadjuvant, in combination with paclitaxel (CS)	Doubled pCR rate in a group preselected for the tumor necrosis factor (*TNF*) gene expression profile	[205]
antisense-miRNA-21 and antisense-miRNA-10b co-delivery	Inhibition of oncogenic miRNAs	Murine models (ES)	reduced tumor growth	[207]
miR-mimic recombinant vectors	Restoration of tumor suppressor miRNAs	Cell line (ES)	Reduced migration and invasion	[209]
Panitumumab	anti-EGFR mAb	Neoadjuvant, in combination with CT	NA	NCT02876107 (phase II) NCT01036087 (phase II)
Apatinib	Anti-EGFR TKI	Advanced, alone or in combination with CT (CS)		NCT05019690 (phase I/II) NCT03932526 (phase II) NCT03254654 (phase II)
Icotinib		Metastatic, pre-treated (CS)	Under evaluation	NCT02362230 (phase II)
Bicalutamide	AR antagonist	Metastatic, AR-positive (CS)	six-month CBR 19%, median PFS 12 weeks	[221]
Abiraterone acetate		Advanced or metastatic, AR-positive pts, in combination with prednisone (CS)	six-month CBR 20.0%, ORR 6.7%, median PFS 2.8 months	[222]
Enzalutamide		Locally advanced or metastatic AR-positive pts (CS)	16 weeks CBR 33%, median PFS 3.3 months, median OS 17.6 months	[223]
Darolutamide		Locally recurrent or metastatic, in AR-positive pts (CS)	NA	NCT03383679 (phase II)

CT: chemotherapy; CS: clinical setting; ES: experimental setting; NA: not available; PARP: polymerase ADP-ribose; RR: response rate; PFS: progression free survival; NTRK: neurotrophic tropomyosin receptor kinase; ORR: overall response rate; Trop-2: trophoblast cell-surface antigen; TGF: tumor growth factor; LRP6: lipoprotein receptor-related protein-6; CSCs: cancer stem cells; PI3K: phosphatidyl inositol 3-kinase; mTORC1/2: mammalian target of rapamycin complex 1/2; GLI: glioma-associated oncogene transcription factor; NFkB: nuclear factor kappa-light-chain-enhancer of activated B cells; mTOR: mammalian target of rapamycin; Akt: protein kinase B; PTEN: phosphatase and tensin homolog; JAK1/2: Janus kinase 1/2; STAT: signal transducer and activator of transcription; DR: death receptor; IAP: inhibitor of apoptosis; SMAC: second mitochondria-derived activator of caspases; TKi: tyrosine kinase inhibitor; AR: androgen receptor; pCR: pathological complete response; CBR: clinical benefit rate; OS: overall survival.

## Data Availability

Not applicable.
